# Working Memory and Its Relation to Deterministic Sequence Learning

**DOI:** 10.1371/journal.pone.0056166

**Published:** 2013-02-08

**Authors:** Markus Martini, Marco R. Furtner, Pierre Sachse

**Affiliations:** Department of Psychology, Leopold-Franzens University of Innsbruck, Innsbruck, Austria; McMaster University, Canada

## Abstract

Is there a relation between working memory (WM) and incidental sequence learning? Nearly all of the earlier investigations in the role of WM capacity (WMC) in sequence learning suggest no correlations in incidental learning conditions. However, the theoretical view of WM and operationalization of WMC made strong progress in recent years. The current study related performance in a coordination and transformation task to sequence knowledge in a four-choice incidental deterministic serial reaction time (SRT) task and a subsequent free generation task. The response-to-stimulus interval (RSI) was varied between 0 ms and 300 ms. Our results show correlations between WMC and error rates in condition RSI 0 ms. For condition RSI 300 ms we found relations between WMC and sequence knowledge in the SRT task as well as between WMC and generation task performance. Theoretical implications of these findings for ongoing processes during sequence learning and retrieval of sequence knowledge are discussed.

## Introduction

Everyday mental activities such as calculating, language comprehension and reasoning require a system that can temporally maintain, flexibly modify and access mental relational representations [1], [2]. This system is called working memory (WM). However, learning to speak in the grammatically correct way or learning properly to walk takes place in an incidental way. This ability to progressively adapt to specific environmental constraints with less or no awareness about how this complex knowledge was acquired and applied is often defined as implicit learning [3], [4]. Incidental sequence learning is a process through which we become sensitive to certain regularities in the environment without intentionally attempting to filter them out.

There are only a few studies linking these two domains whose interactions are currently poorly understood (e.g., [5]). Our study investigated this central issue with WMC tasks representing transformation and coordination [6]–[8] while simultaneously the response-to-stimulus interval (RSI) was varied in a serial reaction time (SRT) task [9], [10]. Next we consider the concepts of WM(C) and incidental sequence learning before we describe the theoretical and empirical connections between them in light of varying the RSI.

### Working memory

WM can be conceptualized as a system that creates and manipulates a limited amount of structural information on the basis of currently activated long-term memory (LTM) units. Within this activated LTM part temporal, direct accessible, bindings between contents (e.g., objects), context (e.g., spatial positions) and procedures (e.g., pressing a specific button) can be set into a new structure and manipulated within the focus of attention [2], [11].

WM capacity (WMC) can be measured by the number of items that can be recalled during a WM task. Most often these WMC tasks require simultaneous storage (maintenance of information, e.g., letters in an active state for an immediate serial recall at the end of a task trial) and processing of a typically unrelated task (e.g., calculating) called complex-span paradigms (e.g., operation span, reading span). Performances in complex-span paradigms are related to a wide range of processing outcomes (e.g., language comprehension, [12]; logic learning, [13]; resisting interference, [14]; suppression of irrelevant or goal incongruent information, [15]; learning new facts, [16]; fluid intelligence, [17]; and integration of preexisting domain knowledge, [18]). However, there is another class of WMC tasks that fall into the category of coordination and transformation tasks [6]–[8]. In these tests participants have to manipulate and/or integrate information to arrive at a correct response. Structural equitation modeling analysis from Oberauer and colleagues [7], [8] showed that relational integration seems to be a function of WM which can be separated from the function of storage and processing. Furthermore, these tasks of coordination and transformation are substantially correlated with a test of reasoning ability and a test of general intelligence [7], [19], [20].

### Incidental sequence learning

It is not a compelling prerequisite to intentionally attempt to learn information. Much of our daily learning is incidental. One of the best paradigms through which to study sequence learning is the SRT task. In a typical SRT task, participants are asked to react to a visual presented stimulus (e.g., a dot). In the deterministic sequence learning task this dot appears sequentially in predefined positions following a repeating pattern and participants have to press a corresponding key as fast and as accurately as possible (vs. probabilistic sequences in which the dot follows a specific sequence only in e.g., 85% of the cases, [21]). Reaction times (RTs) tend to decrease progressively during practice and are increased when the repeating pattern does not follow in the original way [10], [22], [23]. In addition, when learning is incidental, participants acquire more knowledge about the underlying structure than they can consciously convey ([10]; but see [24]).

### Working memory and incidental sequence learning

To describe a possible interaction between WM and sequence learning one has to take into account a possible interaction between WM and LTM. In literature we find increasing theoretical and empirical progress along this line [25], [26]. For Oberauer [2] LTM contributes to the functioning of WM through activation of existing representations and learning and recall of structural information which are represented as chunks. For instance, evidence is found in the Hebb effect that implies that repeated presentation of digit sequences across a series of trials support immediate serial recall of this sequence on further trials even though presentation and recall of other sequences intervenes between these repetitions [27]. Within activated LTM bindings between different representational dimensions can be bound into a new structure, i.e., connections between simultaneously active dimensions are strengthened (e.g., item and position). Long-term learning is possible to accumulate when the input is repeated. During recall then given temporal-contextual cues help to find and reactivate the activation pattern during presentation and therefore the content within LTM [2], [26], [28], [29].

During the binding and learning process the question rises of how much information is under cognitive control, i.e., is explicit and how much information is not under cognitive control, i.e., is implicit. It was argued that incidental sequence learning processes occur with minimal attentional demands [30]. However, this assumption is controversial [31]. Attentional demands on sequence learning are investigated under dual-task conditions. In the dual-task SRT experiment of Nissen and Bullemer [32] either a high- or a low-pitched tone was produced during the RSI, whereby participants were encouraged to count only the low-pitched tones. They argued that the tone-counting task impairs the sequence learning process because of attentional resource exhaustion. Other authors like Stadler [33] argued that a secondary tone-counting task does not limit attentional resources. However, updating processes when encoding a low-pitched tone would insert a temporal asymmetry (extension of the RSI) leading to an inability of the participant to parse the sequence into consistent chunks and consequently impair sequence learning. Frensch and Miner [5], in contrast, argued that short-term memory (STM) limitations are responsible for impaired sequence learning in dual-task conditions. For Frensch and Miner lengthening the RSI leads to decreased sequence learning because it makes it difficult for participants to link together existing memory traces in the activated part of LTM. They found impaired sequence learning with a RSI of 1500 ms compared to a RSI of 500 ms. Furthermore they related STM performance (i.e., digit and location span) to the degree of implicit learning. Their results revealed reliable correlations only when task instructions were intentional or when dual-task situations were realized. Frensch and Miner proposed a theoretical framework for explicit (intentional) and implicit (incidental) sequence learning. They assumed that explicit learning occurs only in the subset of LTM that is in the focus of attention and is achieved through active processing (e.g., hypothesis testing). Implicit learning occurs in the activated subset of STM inside and/or outside the focus of attention and is achieved through a passive associative process. Destrebecqz and Cleeremans [9], [10] investigated the contributions of implicit and explicit knowledge on sequence learning by varying the RSI between 0 ms, 250 ms, and 1500 ms. They found sequence learning in all three conditions but a lack of conscious control in condition RSI 0 and increasing explicit knowledge when RSI was lengthened. Cleeremans and Jiménez [34] and Cleeremans [35] proposed a framework in line with these findings arguing that the difference between explicit and implicit knowledge lays in the quality of the memory traces. Therefore, consciousness about a given representation depends on the strength of the memory traces, their stability in time, and their distinctiveness which is increased by lengthening the RSI. Memory is one fundamental aspect for predicting an upcoming event. This opportunity to generate a prediction might be crucial for learning to occur. Prediction error terms are at the core of many learning models (e.g., [36]), and have been documented in the field of neuroscience (e.g., [37]). Thus RSI might be expected to have an inverted U-shaped relation to sequence learning and WM as well. Smaller RSIs (e.g., 0 ms) should lead to reduced learning because of reduced opportunities to use a prediction and long RSIs (e.g., 1500 ms) should cause a lesser overlap of sequence traces in WM [5], respectively. Strong sequence learning and at the same time possible connections to WM should occur for RSIs that are in between.

### The present study

There are at least two reasons why WM could be included in sequence learning. (1) WM is needed when cognitive control is needed to override automatic response tendencies [26]. In a typical deterministic SRT task several hundred reactions are conducted to a complex training sequence before it is changed to a new sequence. Training leads to a decrease in RTs, change to the new sequence to an increase in RTs. This change is the measure of learned sequence knowledge. When we argue that WM is needed to override automatic response tendencies during the transfer phase, i.e., when LTM representations of the training sequence compete with the new sequence structure for being retrieved then we should find relations between WMC and sequence knowledge. Evidence for this assumption is not found in the sequence learning literature for incidental single task conditions ([5], [38], [39]; the same pattern is found for probabilistic sequences, e.g., [40]). (2) WM is needed to retrieve sequence information from LTM, i.e., for a controlled retrieval of information from LTM information has to be reactivated and captured by the attentional focus [2], [6], [28], [29]. However, it is conceivable that sequence learning takes place outside WM. This is clearly an empirical question. For instance, Keele, Ivry, Mayr, Hazeltine, and Heuer [41] proposed two systems of implicit sequence learning. Sequence learning in the first system is implicit and takes place on noncategorized stimuli within dimensional modules. Sequence learning in the second system can be implicit or explicit and allows associations across dimensions. Thus, the former system would be a theoretical view that makes plausible that WM is not necessarily related to sequence learning.

For the present study two important methodological improvements were made. (1) Studies investigating the relation between WMC and sequence learning most often deployed complex span tasks (beside simple span tasks) which depicted only the WM function of maintaining information in a highly active state and overcome competition when processing a secondary task [5], [38]–[40]. We believe using only this class of tasks narrows the construct and function of WM. For this we used two validated tasks namely memory updating (MU) and spatial short-term memory (SSTM) taken from the WM test battery from Lewandowsky, Oberauer, Yang, and Ecker [20]. With the applied WM measures the demands on WM of storage and replacing old WM contents by new contents (MU task) and relational integration, i.e., building of new relations between elements (SSTM task, [7], [20]) were measured. (2) MU and SSTM task scores were related to sequence knowledge in the SRT task and performance in the followed free generation task of two RSI conditions (RSI 0 ms vs. RSI 300 ms).

Based on the theoretical outlines above we predicted sequence knowledge in both RSI conditions and higher correlations with WMC scores in condition RSI 300. High WMC individuals should acquire more sequence knowledge than low WMC individuals. For the following free generation task we predicted higher generation scores in condition RSI 300. High WMC individuals should generate more sequence knowledge than low WMC individuals.

## Methods

### Ethics Statement

The present research was conducted with approval by the Institutional Review Board of the University of Innsbruck (Faculty of Psychology and Sport Science). The data were analyzed anonymously. Participants gave written informed consent.

### Participants

Fifty-eight undergraduate students (14 males, 44 females, mean age = 21.21 years, *SD* = 1.77, range = 19–27 years) took part in the experiment. None of them had previously taken part in any sequence learning experiment. They were randomly assigned to condition RSI 0 ms (*n* = 25) and RSI 300 ms (*n* = 33).

### Material and Procedure

The three tasks were MU, SSTM, and SRT. The tasks were always administered in this order. The MU and SSTM tasks took together approximately 30 minutes. They were taken from the WM test battery of Lewandowsky, Oberauer, Yang, and Ecker [20] and run on MATLAB 7.10.0.499 (R2010a) and Psytoolbox Version 3 [42], [43]. The sequence learning and generation task took about 20 min and was programmed in Java Version 6. All four tasks run on laptops with 14-inch screens.

#### MU task

Participants had to hold in mind an initial set of digits, each presented in a separate frame on the screen, and to subsequently update these digits through arithmetic operations. This task represents updating because information has to be retrieved, transformed, and substituted [44]. Set size varied between three to five frames per trial (frames were organized horizontally, whereby three frames were presented in one row; four and five frames were presented in two rows). After a key press the starting digits were presented in their frames, one by one, for 1 sec each. Arithmetic operations (ranging between −7 to +7, excluding 0) were displayed in individual frames for 1.3 sec. each, followed by a 250 ms blank interval. The participants had to apply the operation to the digit that they currently remembered in that frame and were instructed to replace the memorized content by the result. The final recall was signaled by question marks appearing one by one in each frame. The interim and final results ranged from 1 to 9. Participants were instructed to type the remembered digit for that particular frame. There was no time constraint for recall, and no performance feedback was provided. The 15 test trials were preceded by two practice trials. Digits, operations, updating, recall orders, and trial orders were generated randomly.

#### SSTM task

Participants had to remember the location of two to six dots per trial in a 10×10 grid. Following the central presentation of a fixation cross for 1 sec, the grid was shown, and randomly generated dots appeared, one by one, in the cells of the grid for 900 ms each (interstimulus interval 100 ms). The participants were instructed to remember the spatial relations between the dots. Absolute dot positions were irrelevant; only the overall pattern of dots was to be remembered. After all of the dots were presented, the participants had to reproduce the pattern of dots in a blank grid of the same size by clicking the cells with the computer mouse. No feedback was given. There were 30 trials, 6 at each set size. Test trials were preceded by two practice trials. The SSTM score was computed according to the distance between the learning dots and the generated dots. If the distance is 0 cells, 2 points are gained. If the distance is 1 cell, 1 point is gained. If the distance is further than 1 cell, 0 points are gained. The total score in the SSTM task is the sum of all scores on all trials [20]. The SSTM task is not an updating task because the dot positions needed not to be transformed and substituted. Furthermore the SSTM task requires more spatial abilities than the MU task because stimuli are presented within a frame consisting of 100 cells.

#### SRT task

The display consisted of four small grey orientation dots arranged in a horizontal line on the computer's screen. Orientation dots were separated by intervals of 3 cm. Each screen position corresponded to a key on the keyboard. The spatial configuration of the four keys was fully compatible with the four screen positions. The stimulus was a small black circle that appeared on a white background. On each trial, this stimulus appeared at one of four possible screen locations. Participants were instructed to respond as fast and as accurately as possible by pressing one of the four possible keys marked as response keys. The target was removed as soon as a key was pressed, and the next stimulus appeared after either a 0 ms (condition RSI 0) or 300 ms (condition RSI 300) delay. Response latencies were measured from the onset of the target to the completion of a correct response. The target followed one of the two second-order conditional sequences (SOC 1 = 3-4-2-3-1-2-1-4-3-2-4-1; SOC 2 = 3-4-1-2-4-3-1-4-2-1-3-2). In SOC sequences every location is completely determined by the previous two locations. They are balanced for location frequency (each location occurred three times), transition frequency (each location was preceded once by each of the other three locations), reversal frequency (e.g., 1-2-1, one in each sequence), and they have no repetitions [45]. The two SOC's only differed in terms of the subsequence of the three elements that they contained (e.g., the transition 3–4 was followed by locations 2 in SOC 1 and by locations 1 in SOC 2). The whole SRT task consisted of 10 blocks for a total of 960 trials. Short rest breaks occurred between each block. Participants were trained on SOC 1 during blocks 1–8 and 10, and on SOC 2 during block 9. After the SRT task, subjects were informed that the dots had followed a repeating pattern. Participants then were instructed to freely generate a series of 96 trials that resembled the training sequence as much as possible. They were told to rely on intuition when feeling unable to recollect the location of the next stimulus and to find back into the response rhythm they followed during the training phase.

## Results

### SRT task

**Figure 1:**
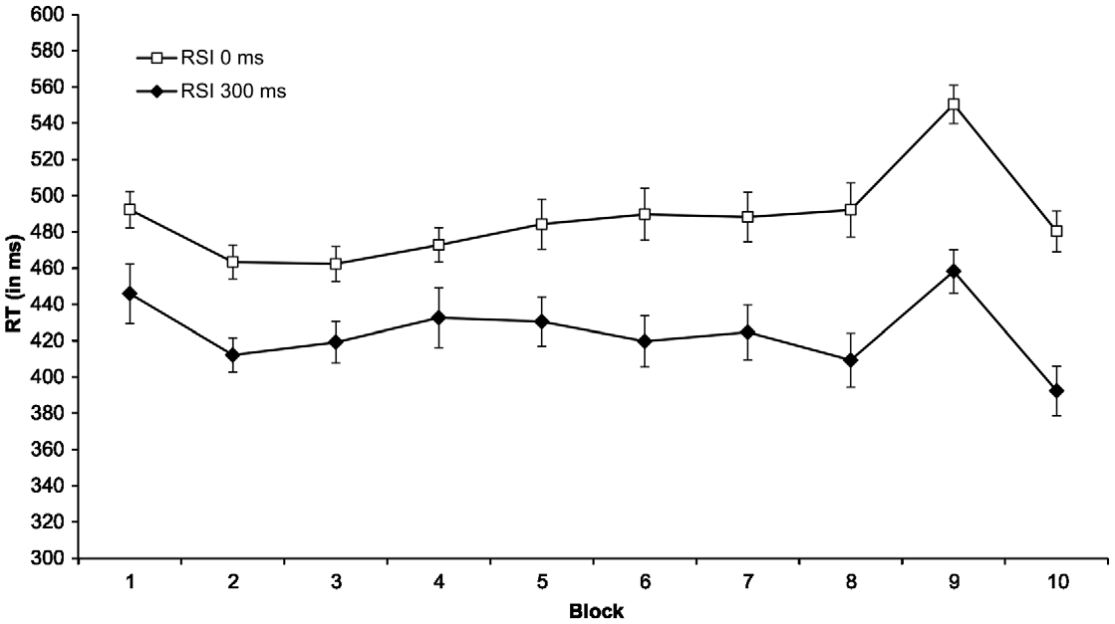
Mean RTs by RSI conditions. Mean RTs (in ms) for training blocks (1–8 and 10) and transfer block (9), plotted separately for the corresponding RSI conditions. Error bars depict standard errors.

The RT analysis was performed on the participants mean correct RTs. Mean RTs were calculated for each participant and each block after removal of erroneous responses, discard of the first two trials from each block, and correct responses with RTs three standard deviations above the mean RT of the block (see Figure 1, Table 1). Three outliers were removed from analysis in condition RSI 300 (*n* = 30). Overall error rates were assessed by dividing the number of incorrect target localization responses by 940 trial responses. Error rates were low in both conditions (RSI 0: *M* = .06, *SD* = .04; RSI 300: *M* = .05, *SD* = .02). A *t*-test revealed no significant differences, *t*(34.20) = .96, *p* = .350. Analysis of training block 8 and transfer block 9 revealed higher error rates in block 9 in both conditions (see Table 1). We calculated a difference score of errors (De score) of the proportion of errors in block 9 and block 8 for condition RSI 0 (*M* = .04, *SD* = .06) and RSI 300 (*M* = .01, *SD* = .04) and found a significant difference, *t*(53) = 2.39; *p* = .020.

**Table 1:** Descriptive statistics of the relevant data for the two RSI conditions.

		Mean RTs Block 8	% errors Block 8	Mean RTs Block 9	% errors Block 9	Mean RTs Block 10	% errors Block 10	MU	SSTM	D	De	G
RSI 0	*M*	492.09	6.2	550.41	10.5	480.28	8.5	.51	.82	58.32	.04	.38
	*SD*	74.56	7.3	52.94	9.7	56.45	10.6	.15	.04	57.55	.06	.11
RSI 300	*M*	409.31	5.9	458.22	6.8	392.34	5.4	.57	.84	48.91	.01	.46
	*SD*	81.27	3.2	65.51	3.4	74.69	2.9	.17	.05	49.98	.04	.13

*Note*: MU = memory updating, SSTM = spatial short-term memory, D = difference score, De = difference score of the errors, G = generation score.

In order to analyze the RT data, we performed a mixed-design ANOVA with block (1–10) as within-subject variable and condition (RSI 0 vs. RSI 300) as between-subjects variable. Greenhouse-Geisser corrected analysis revealed significant effects of block (*F*(5.20, 275.54) = 16.20, *MSE* = 2266.13, *p*<.001) and condition (*F*(1, 53) = 15.20, *MSE* = 35744.67, *p*<.001). Moreover, a significant block×condition interaction was found indicating superior learning in condition RSI 300 (*F*(5.20, 275.54) = 3.86, *MSE* = 2266.13, *p* = .002).

To assess sequence knowledge, we conducted a second mixed-design ANOVA with block (8 and 9) as within-subject variable and condition (RSI 0 vs. RSI 300) as between-subjects variable. The reason for involving block 8 and not the mean RTs of blocks 8 and 10 were our interest in the direct switch from a highly learned training phase to a new sequence in the transfer phase. The mean RTs of blocks 8 and 10 would include RTs from the switch of a weakly learned sequence structure (block 9) to a trained one (block 10). Analysis revealed significant effects of block (*F*(1, 53) = 54.67, *MSE* = 1433.91, *p*<.001) and condition (*F*(1, 53) = 25.11, *MSE* = 8314.79, *p*<.001). The block×condition interaction was not significant (*F*(1, 53) = .42, *MSE* = 1433.91, *p* = .520). These results indicate that participants acquired equal amounts of sequence knowledge in both conditions, and the transfer effect is not significantly influenced by the value of the RSI.

### Generation task

To assess generation performance, we computed the number of generated chunks of three elements that were part of the training sequence (G score). The generated knowledge test (see Table 1) of the training sequence consisted of 96 trials. The maximum number of correct chunks that could be produced was 94. G score was therefore calculated for each person as the sum of triplets divided by 94. Two one sample *t*-tests were computed to compare generation scores to chance level (chance level was .33; because no repetitions were allowed, only three options remained after each key press; e.g., [9], [10]). G scores were significantly above chance level in condition RSI 0 (*t*(24) = 2.48, *p* = .020) and RSI 300 (*t*(28) = 5.44, *p*<.001). These results indicate that learning was explicit to some extent in both conditions. Furthermore, G scores in condition RSI 0 and RSI 300 differed significantly (*t*(52) = −2.44, *p* = .020), indicating that more explicit knowledge was generated in condition RSI 300 compared to RSI 0.

### Correlational analysis between working memory capacity, RT measure of sequence knowledge, error indicator of sequence knowledge and generation measure of sequence knowledge

In order to analyze the relation between WMC, and the three measures of sequence knowledge (RT, error, and generation knowledge) we computed bivariate zero-order Pearson correlations. As a RT measure of sequence knowledge we used the above outlined D score (difference of the mean RT of transfer block 9 and training block 8). In regard to the error indicator of sequence knowledge we applied the above calculated De score (proportion of errors in transfer block 9 minus the proportion of errors in training block 8). As a generation measure of sequence knowledge we used the above mentioned G score (number of triplets/94). Scores of WMC measures (MU and SSTM) were then related to D, De, and G scores for condition RSI 0 and RSI 300. Furthermore for testing the difference between two correlation coefficients of condition RSI 0 and RSI 300 we conducted a Fisher's r-to-z transformation.

#### D scores

We found no significant correlations between WMC measures with D scores in condition RSI 0 (MU: *r* = .06, *p* = .770; SSTM: *r* = .10, *p* = .630) and significant correlations in condition RSI 300 (MU: *r* = .50, *p* = .005; SSTM: *r* = .46, *p* = .010). These results indicate that the higher the MU score and the higher the SSTM score, respectively, the higher is the difference of the mean RT of block 8 and 9 in condition RSI 300. In addition the difference among the correlation coefficients between condition RSI 0 (r_MU-D score_ = .06; r_SSTM-D score_ = .10) and RSI 300 (r_MU-D score_ = .50; r_SSTM-D score_ = .46) were significant for MU (*z* = −1.70, *p* = .045, one-tailed) but not for SSTM (*z* = −1.38, *p* = .084, one-tailed). The significant result indicates a higher correlational coefficient in condition RSI 300.

#### De scores

One significant correlation was found for De scores in condition RSI 0 (MU: *r* = −.17, *p* = .432; SSTM: *r* = −.44, *p* = .027) and no correlations with De scores in condition RSI 300 (MU: *r* = .03, *p* = .883; SSTM: *r* = −.26, *p* = .162). These results indicate that the higher the SSTM score, the lower is the difference of the proportion of errors in block 8 and 9. The difference among the correlation coefficients between condition RSI 0 (r_MU-De score_ = −.17; r_SSTM-De score_ = −.44) and RSI 300 (r_MU-De score_ = .03; r_SSTM-De score_ = −.26) revealed no significant differences for MU (*z* = −.68, *p* = .248, one-tailed) and SSTM (*z* = −.72, *p* = .235, one-tailed).

#### G scores

We found no significant correlations between WMC measures with G scores in condition RSI 0 (MU: *r* = −.21, *p* = .320; SSTM: *r* = −.11, *p* = .610) and significant correlations in condition RSI 300 (MU: *r* = .53, *p* = .003; SSTM: *r* = .58, *p* = .001). These results indicate that the higher the MU score and the higher the SSTM score, respectively, the higher is the number of generated triplets. The difference among the correlation coefficients between condition RSI 0 (r_MU-G score_ = −.21; r_SSTM-G score_ = −.11) and RSI 300 (r_MU-De score_ = .53; r_SSTM-De score_ = .58) revealed a significant difference for MU (*z* = −2.77, *p* = .003, one-tailed) and SSTM (*z* = −2.67, *p* = .004, one-tailed). These results indicate significantly higher correlational coefficients in condition RSI 300.

## Discussion

The present study adds to a small number of studies so far to examine relationships between WM and sequence learning. Overall results show that WM is involved in sequence learning and generation of explicit knowledge about an underlying structure when RSI is lengthened. Furthermore, we found a relation between WM and an error indicator of sequence knowledge when there is no-RSI. Our results indicate that a higher WMC might predict better learning and less impact of trained sequence knowledge simultaneously. High WMC individuals might show more learning and are potentially better able to shield retrieval of misleading sequence knowledge in the block with the novel sequence. To additionally test the learning prediction we took the difference of the mean RT of the first training block and the last pre-transfer block as indicator of sequence learning. This difference should be unaffected by effects of inhibition of misleading sequence knowledge which might come to effect in the transfer block. Results of Pearson correlational analysis confirmed this prediction for condition RSI 300 (MU: *r* = .53, *p* = .002; SSTM: *r* = .39, *p* = .034; RSI 0: MU: *r* = .04; *p* = .868; SSTM: *r* = .12; *p* = .563). However, this analysis might not only capture RT decreases by sequence learning but might also include attentional and motivational variations. Significant RT increase in the block with the novel sequence can be the result of (a) a large amount of sequence knowledge (b) low capacity to shield performance from retrieval of misleading sequence knowledge, or both. In addition, the generation task performance (G score) and the learning gain (first training block minus last pre-transfer block) should both not be substantially influenced by the capacity to shield from misleading sequence knowledge (transfer block minus last pre-transfer block, D score). Pearson correlational analysis revealed for condition RSI 0 a significant relation between D score and learning gain (*r* = .68, *p*<.001) and no significant correlation between D score and G score (*r* = .03, *p* = .880). For condition RSI 300 we found no relations between D score and learning gain (*r* = .30, *p* = .114) and a significant correlation between D score and G score (*r* = .59, *p* = .001).

During learning of a repeating sequence a LTM representation of this sequence is generated. Repetition of the sequence leads to stronger LTM representations and therefore more sequence knowledge. This notion is in line with results of studies on immediate serial recall indicating that background knowledge concerning domain-specific regularities in sequential structure can affect recall performance. For instance Baddeley, Conrad, and Hull [46] could show that consonant strings were better recalled when they contained high-frequency letter transitions compared to low ones. Furthermore in an incidental phonological learning paradigm participants of Majerus, Van der Linden, Mulder, Meulemans, and Peters [47] listened to a continuous sequence of stimuli that was based on an artificial phonotactic grammar. Their results could show that verbal STM performance was improved for lists that conformed to the artificial grammar. During the transfer block then active LTM representations compete for being selected into the focus of attention [2], [28]. LTM knowledge of the training sequence discussed here can affect response decisions in the transfer block. The stronger the representation of the training sequence is the more it will interfere with the new sequence in the transfer block. Higher WMC scores relate to stronger training sequence representations but at the same time these representations affect adaption to a new sequence at a greater extent which resulted in higher D scores.

The influence of LTM sequence representations may also be responsible for the unexpected high error rates in the transfer block compared to the last training block in condition RSI 0. Botvinick and Bylsma [48] tested participants on immediate serial recall on sequences of pseudowords generated on the basis of an artificial grammar. They could show that errors display a tendency toward regularization that is a bias toward sequences which are higher in probability than the actual, to be recalled stimulus. Furthermore investigations into low versus high WMC individuals indicate that low WMC individuals are more error prone (e.g., antisaccade task, [49]; Stroop task, [15]; or in shadowing during a dichotic listening task, [50]). We hypothesize that low WMC individuals in condition RSI 0 have a higher tendency to regularization compared to high WMC individuals.

Results of the generation task revealed that lengthening the RSI resulted in a higher proportion of explicit knowledge, i.e., higher controllable sequence knowledge compared to the no-RSI condition. Despite the fact that no difference in sequence learning performance itself existed, the formed representations during sequence learning in the RSI condition were of higher accessibility for retrieval processes from LTM into the focus of attention. This may be accomplished by a finer tuned adjusting of thresholds within active LTM representations during training blocks for later retrieval [2], [28], [51]. During sequence learning specific stimulus-response representations are set and connected based on an underlying sequence structure. When it comes to a switch to a new sequence the old sequence competes for being retrieved into the focus of attention. Low set thresholds in the training phase of condition RSI 0 might therefore lead to a higher old-new sequence competition during the transfer block. Low WMC individuals were less able to shield performance from retrieval of the old sequence leading to higher error rates. The same picture can be drawn for condition RSI 300. Here, WM was significantly involved in higher training phase learning gain (block 1 minus block 8) setting higher dissociable thresholds. Furthermore WM was significantly involved in the switch from the old sequence structure to a new one in the transfer block. High WMC individuals therefore learned more and had to shield performance from misleading sequence knowledge to a bigger extent than low WMC individuals. These higher set thresholds in condition RSI 300 might be responsible for the difference of explicit knowledge in the generation task. During retrieval in the generation task activated LTM representations continuously compete for being retrieved into the focus of attention [2]. The stronger and dissociable the threshold signals are, the higher the probability that sequence parts are correctly retrieved into the focus of attention. In condition RSI 0 these thresholds seemed to be set too low which might led to concurring co-activated sequence parts. In condition RSI 300 higher thresholds seemed to be set during sequence learning of high WMC individuals which led to significantly higher generation scores for high WMC individuals compared to low WMC individuals. These qualitative different representations seem to differentiate them between implicit and explicit knowledge [34], [35] and are in line with a recent WM theory of Oberauer [2] who described two interacting processing modes, an analytic processing and an associative mode that are seen as the end points of a continuum. The associative system consists of the activated part of the LTM whereas the analytic mode represents more an active state that ideally excludes influences from active LTM and which enables selection and manipulation of those representations. It is conceivable that incidental sequence learning takes place in the associative processing mode where specific WM processes adjust the threshold on the LTM sequence representations. When there is a transition from the trained sequence to a new sequence the old one competes for being retrieved in the attentional focus ([2] termed it response focus in the procedural WM). For Oberauer [2] the retrieval from LTM is automatic, without being intended. In our case of sequence learning this means that stronger LTM representations should have a privileged status of being retrieved into the attentional focus compared to weaker representations that are formed in the transfer block. The strength of sequence representation together with the instructional goal for reacting on the appearing stimulus as accurately and fast as possible produces a conflicting situation. In this situation the higher the acquired sequence knowledge is at the end of the training blocks the more capacity it takes to shield performance of misleading LTM sequences representations for being retrieved during the transfer block. The associative system should play a role in the generation task where thresholds for retrieval from LTM have to be raised. The higher the threshold is, the weaker the influence of co-activated LTM representations and the higher the probability of correctly retrieving the sequence of the training phase or parts of it.

Our study can show that WM is related to deterministic sequence learning. However some critical points have to be addressed. First, it is conceivable that RSI affects the acquisition of sequence knowledge and/or the employment of sequence knowledge to boost performance. In order to disentangle effects of RSI on learning versus performance one should equate RSI between conditions when testing for sequence knowledge at the end of the sequence learning task. A similar structural problem has been raised by Frensch and colleagues [30], [52] in the debate on secondary task load. Their studies suggest that it is not sequence learning per se that is affected by the secondary task. Rather, sequence learning is suppressed by the secondary task. After the concurrent task was removed in the transfer phase results indicated that equal amounts of sequence knowledge were learned. Second, we placed our focus primarily on the relation between WM and sequence learning from a capacity perspective. Considering the content perspective Abrahamse, Jiménez, Verwey, and Clegg [53] suggested that task sets held in WM determine the content of sequence knowledge. Gaschler, Frensch, Cohen, and Wenke [54] put this prediction to test in a SRT task by varying whether the mapping of gray shapes to keys was instructed based on the color or based on the position of keys. Their results suggested that instructions influenced how participants coded responses in WM and which content they acquired in sequence learning. While participants in the color instruction condition acquired response color sequence knowledge, participants in the spatial instruction condition acquired response position sequence knowledge.

Future research has to further clarify the role of the relations between WM and sequence learning. This might include exploring similarities and differences in the representation of serial order in WM and implicit sequence learning [cf. 55] and testing how representations of serial order in WM might translate to the representation of serial order in implicit sequence knowledge. For example, for Oberauer [28] two long-term learning mechanisms exist, namely chunking of structures and the gradual build-up of associations. Evidence of the former comes from analogical retrieval (e.g., [56]), the Hebb effect (e.g., [27]) and retrieval times for lists (e.g., [57]). Evidence of the latter comes from varying the transition probabilities of an item sequence based on an artificial grammar which results in gradual learning of frequently repeated trials [29], [46], [58], [59]. Schuck, Gaschler, Keisler, and Frensch [55] discuss work from the verbal learning tradition (including the Hebb effect) on the representation of serial order and models on the representation of serial order in WM in order to then test the representation of serial order in implicit sequence learning. Based on our results it might be a next step to directly test whether and how the presentations of serial order in WM determines the presentation of serial order in implicit sequence knowledge, i.e., does implicit sequence learning store the order representation held in WM? This is not necessarily the case. There are evidences outside the sequence learning literature that assume that binding of features in WM did not lead to binding of features in LTM (e.g., [60], [61]). In addition, further fundamental executive functions should be identified that might be involved in sequence learning (e.g., inhibition, [15]; focus switching, [62]; and lower level control processes such as response selection, [63]).
